# Therapeutical Notes

**Published:** 1900-04-07

**Authors:** 


### THERAPEUTICAL NOTES.

### Chronic Bronchitis.

Neusser recommends the following:—

℞. Acidi carbolici, 0·5.

Icthyol, 5·0.

Sp. vini, 10·0.

Aq. distillatæ, 100·0.

Misce fiat inhalatio.

—*New York Med. Jour.*, November 11th.

### Tender Feet.

Major Macpherson refers to many methods of treatment. The foot-powder of the German army consists of salicylic and chromic acids, which act as germicides and harden the epidermis. A common remedy consists in soaking the feet and stockings in boric acid from time to time. Offensive feet are best treated by washing the inside of the boots and socks, the soles of the feet, and between the toes with a 2 per cent. solution of formaline. Adler (quoted by Merck) points out that in some persons formaline causes fissures between the toes. In such cases Macpherson would apply tannoform, which contains sufficient formaldehyde to disinfect the skin while it keeps the surface dry and sound.—*Practitioner*, December.

### Gonorrhœal Epididymitis.

Pigot (*Thèse de Paris*, March 11th) reports very good results in 32 cases treated by the internal administration of salicylate of soda. No other treatment is, he thinks, so useful as this when the cord is not greatly affected, and where there is only slight effusion into the tunica vaginalis. If, however, the cord is much involved inunction of mercury and belladonna must be employed. —*Merck's Archives*, December.

### Uncontrollable Hæmorrhage.

Injections of gelatine solution may be given to increase the coagulability of the blood and to prevent bleeding in gastric ulcer, typhoid, phthisis, or hæmophilia. R. Heyman, after a tonsillotomy, injected 140 grammes of a 2½ per cent. solution under the skin of the chest, which stopped the bleeding. The effect, however, is only temporary, and in this case the injections had to be repeated on the next two days. Complete aseptic precautions should be observed.—*Merck's Archives*, December, 1899.

### Otitis Media.

In profuse suppuration Grüber employs astringents in gelatine bougies, each of which contains zinc sulphate, copper sulphate, or sodium borate. In badly-smelling cases similar bougies containing iodoform are used. Each bougie has from ·15 grain to ·7 grain of the remedy.—*Landesman, Die Therapie an den Wiener K.*

### Incontinence of Urine.

A troublesome form is seen in elderly females where the water runs away in the act of sneezing, coughing, or laughing, which is due to want of power in the sphincter vesicæ. One minim of the tincture of cantharides given in water three or four times a day will be found of the greatest service.—*Med. Press and Circ. and New York Med. Jour.*, November 25th.

### Strontium Lactate in Albuminuria.

S. Bronowski (*Wiener Medicinische Presse*, 1899, Num. 5) finds that this drug causes a general lowering of blood pressure. Moderate doses have a diuretic effect, probably by dilating the vessels of the kidneys without any action on the epithelium. A reduction of the amount of albumen passed is observed in most cases of Bright's disease, unless they have reached an advanced stage.—*New York Med. Jour.*, November 25th.

